# Optimization of an anatomically and electrically detailed rodent subthalamic nucleus neuron model

**DOI:** 10.1152/jn.00287.2023

**Published:** 2024-06-12

**Authors:** Hengji Chen, M. Sohail Noor, Clayton S. Bingham, Cameron C. McIntyre

**Affiliations:** ^1^Department of Biomedical Engineering, Duke University, Durham, North Carolina, United States; ^2^Department of Neurosurgery, Duke University, Durham, North Carolina, United States

**Keywords:** computational modeling, deep brain stimulation, genetic algorithm, ion channels

## Abstract

Deep brain stimulation (DBS) of the subthalamic nucleus (STN) is an effective treatment for Parkinson’s disease, but its mechanisms of action remain unclear. Detailed multicompartment computational models of STN neurons are often used to study how DBS electric fields modulate the neurons. However, currently available STN neuron models have some limitations in their biophysical realism. In turn, the goal of this study was to update a detailed rodent STN neuron model originally developed by Gillies and Willshaw in 2006. Our design requirements consisted of explicitly representing an axon connected to the neuron and updating the ion channel distributions based on the experimental literature to match established electrophysiological features of rodent STN neurons. We found that adding an axon to the STN neuron model substantially altered its firing characteristics. We then used a genetic algorithm to optimize biophysical parameters of the model. The updated model exhibited spontaneous firing, action potential shape, hyperpolarization response, and frequency-current curve that aligned well with experimental recordings from STN neurons. Subsequently, we evaluated the general compatibility of the updated biophysics by applying them to 26 different STN neuron morphologies derived from three-dimensional anatomical reconstructions. The different morphologies affected the firing behavior of the model, but the updated biophysics were robustly capable of maintaining the desired electrophysiological features. The new STN neuron model developed in this work offers a valuable tool for studying STN neuron firing properties and may find application in simulating STN local field potentials and analyzing the effects of STN DBS.

**NEW & NOTEWORTHY** This study presents an anatomically and biophysically realistic rodent STN neuron model. The work showcases the use of a genetic algorithm to optimize the model parameters. We noted a substantial influence of the axon on the electrophysiological characteristics of STN neurons. The updated model offers a valuable tool to investigate the firing of STN neurons and their modulation by intrinsic and/or extrinsic factors.

## Graphical abstract

**Figure:**
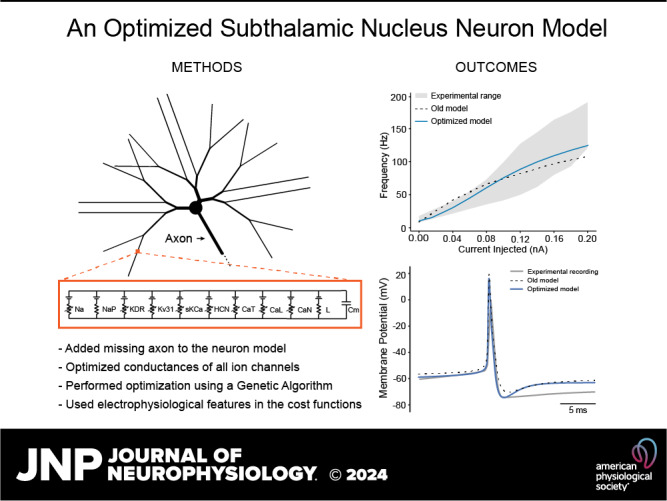

## INTRODUCTION

Deep brain stimulation (DBS) of the subthalamic nucleus (STN) is an established treatment for advanced Parkinson’s disease (PD) (1). Although STN DBS can alleviate the motor symptoms of PD, its mechanisms of action remain unclear (2). Therefore, a need exists for model systems that can simulate STN neuron activity and provide a testing ground for DBS research. Multicompartment cable models of STN neurons are commonly used in this regard (3).

The current standard for a detailed rodent STN neuron model was established by Gillies and Willshaw (GW) in 2006 (4). The GW model captures many important features of STN neurons and has been successfully used in many different research studies. However, the GW model has several shortcomings. First, it lacks an axon, which is known to play a crucial role in the neural activity and spiking dynamics of STN neurons (5). The axon plays an even more important role in DBS modeling, as it is the site of action potential initiation from extracellular electric fields (6). Second, the ion channel conductance parameters used in the GW model merit revision to better align with experimentally determined values. For example, their use of channel density gradients along the dendrites is not supported by the established literature (7–10). In addition, three ion channels (CaL, HCN, and KDR) in the GW model have higher than expected density in the distal region of the dendrites (7, 8, 11).

Given the limitations of the GW model, we aimed to develop an updated rodent STN neuron model that would be more anatomically and biophysically realistic. First, we explicitly incorporated an axon into the neuron model. Second, we constrained the ion channel distributions to better coincide with the existing literature. Then, we optimized the conductance parameters of the model using a genetic algorithm that evolved to match metrics derived from experimental recordings. Finally, we assessed the compatibility of the updated biophysics by applying them to 26 different STN neuron morphologies and evaluated the resulting electrophysiological behaviors.

## METHODS

**Figure 1.**
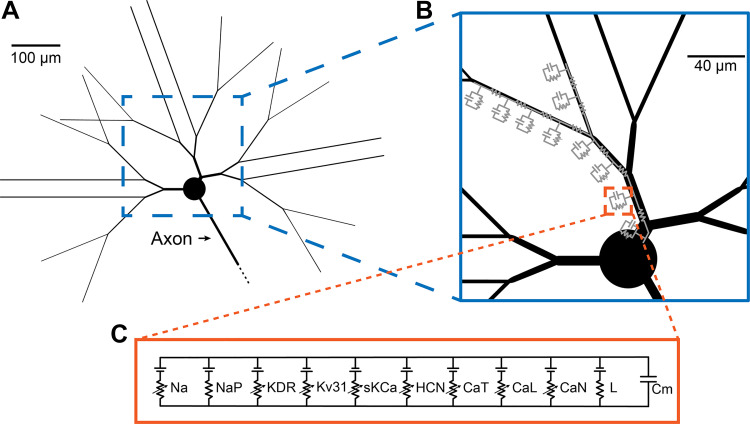
Multicompartment model of a rodent STN neuron. *A*: morphology of the STN neuron model. *B*: the soma and dendrites were segmented, and each segment was modeled as a capacitor and multiple conductances connected in parallel. *C*: ion channels, passive conductance (L) and membrane capacitance (Cm) were present in all compartments. CaT, low voltage-activated calcium channel; CaL, high voltage-activated calcium channel; CaN, high voltage-activated calcium channel; HCN, hyperpolarization-activated cation channel; KDR, potassium delay rectifier; Kv31, potassium fast rectifier; Na, transient sodium; NaP, persistent sodium; sKCa, small conductance calcium-activated potassium channel; STN, subthalamic nucleus.

**Figure 2.**
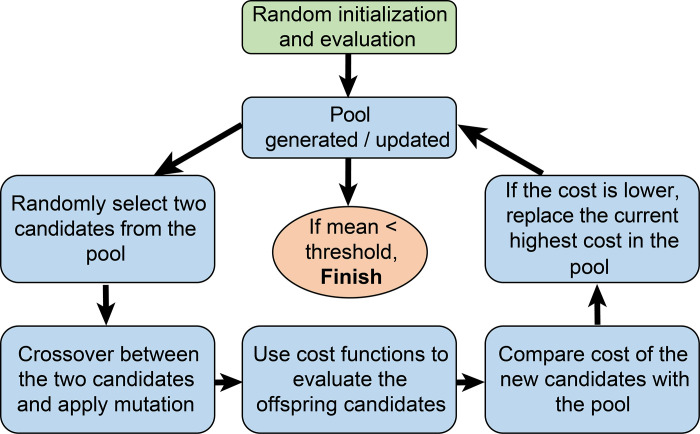
The flow chart of the genetic algorithm used in the optimization.

We developed our updated rodent STN neuron model in the NEURON simulation environment (12) and used a genetic algorithm to optimize its biophysical parameters (13, 14) (Fig. 1). Genetic algorithms have become a common approach for neuron model parameterization and they consistently produce promising results (15–17). In this study, we used a custom genetic algorithm and cost functions to optimize the STN neuron model (Fig. 2).

To stay consistent with the original GW model, we performed our optimization within the original GW STN soma-dendritic neuron morphology (4) (Fig. 1). We then added an axon to that morphology (3). The basic channel parameters also remained consistent with the original GW model (4). However, we optimized the passive membrane properties, as well as the cytoplasmic maximum conductance values of the active ion channels in both the soma and the dendrites. The genetic algorithm was tasked with optimizing the model parameters to match four common electrophysiological features (Fig. 2): *1*) input impedance, *2*) spontaneous spiking rate, *3*) the shape of the action potential, which includes the peak, the afterhyperpolarization potential, and the half spike width, *4*) the frequency-current (F-I) curve, and *5*) response to a hyperpolarization current injected into the soma.

### Axon Compartments

We added a myelinated axon to the GW model (Fig. 1). The axon morphology and biophysics were adopted from a previous primate STN neuron model (3), which were derived from the standard MRG model of a myelinated axon (18). Our updated rodent STN neuron model added an axon initial segment (AIS) of 1.89 μm diameter, 10 nodes of Ranvier of 1.4 μm diameter, and myelinated internodal compartments of 2 μm outer diameter. The axon was connected to the soma through the AIS.

### Biophysics and Ion Channel Distributions

The complete STN neuron model consisted of a soma, three primary dendrites with a total of 18 dendritic tips, and an axon (Fig. 1*A*). The soma, dendrites, and axon were segmented into compartments, where each compartment was represented by the combination of a capacitor, a passive conductance, and active conductances, all connected in parallel (Fig. 1*B*). The active conductances represented various ion channels while the passive conductance accounted for membrane leakage. For the soma and dendrites, we used the same set of nine active ion channels used in the original GW model (Fig. 1*C*). A key goal of this study was to optimize those channel conductance values.

The passive membrane properties of the model are shared between the soma and dendrites, as setup in the original GW model (4). The active ion channels were allowed to have different conductance values in the soma compared with the dendrites. However, the active ion channel density gradients that were present in the original GW model were removed, and all channels were made uniformly distributed on the dendrites. Six channels were exclusively present on the soma and proximal dendrites while the remaining three channels were distributed across all somatic and dendritic compartments (Table 1). Dendritic compartments located within a distance equal to or less than half of the maximum dendrite path length were considered proximal. For the axon, only the AIS sodium channel conductance was optimized, and all other aspects of the axon model were held constant with their original implementation (3).

**Table 1.** Ion channel conductance range and location on dendrites

Ion Channel	Location	Conductance Range in Soma, S/cm^2^
Na (transient sodium channel)	Soma and proximal dendrites (10)	3e-3 ∼ 4e-2 (11, 19)
NaP (persistent sodium channel)	Soma and proximal dendrites (10)	0 ∼ 2e-4 (4, 20)
CaL (high voltage-activated calcium channel)	Soma and proximal dendrites (7)	0 ∼ 2e-3 (4, 21)
CaN (high voltage-activated calcium channel)	All somatic and dendritic compartments (22)	0 ∼ 2e-3 (11)
CaT (low voltage-activated calcium channel)	Soma and proximal dendrites (23)	0 ∼ 5e-3 (4, 21)
sKCa (small conductance calcium-activated potassium channel)	All somatic and dendritic compartments (9)	0 ∼ 5e-4 (4, 11)
Kv31 (potassium fast rectifier)	Soma and proximal dendrites (24)	5e-3 ∼1.5e-1 (4, 11)
KDR (potassium delay rectifier)	Soma and proximal dendrites (8)	5e-4 ∼ 5e-3 (4)
HCN (hyperpolarization-activated cation channel)	All somatic and dendritic compartments (11)	0 ∼ 2e-3 (11)
Na in AIS		10 ∼ 30 (25, 26)

Based on the available literature, the conductance density of most ion channels (except CaN, CaT, and HCN) is generally lower or uniform in the dendrites when compared with the soma (11, 27–29). Therefore, we optimized the dendritic conductance density by determining scaling factors for each individual channel. These scaling factors, ranging from 0 to 1, were optimized and then multiplied by the optimized somatic conductance density to drive the corresponding dendritic conductance. Conductance density of the HCN channel was defined to be the same in both the soma and dendrites (11). The conductances of CaN and CaT channels in dendrites were not constrained by their corresponding somatic conductances. In total, we optimized 20 different maximum conductance values, nine active conductances specific to the soma, eight active conductances specific to the dendrites, one active conductance for the AIS, and two passive conductances.

### Optimization Algorithm and Cost Functions

We used a genetic algorithm written in Python to optimize all 20 parameters mentioned in the previous section (Fig. 2). The algorithm generated ∼1,000,000 phenotypes using crossover and mutation and selected the best candidates by evaluating cost functions. The optimization process started with the algorithm initializing a pool of 120 individual models by randomly picking conductance values for each ion channel from the given range. The algorithm then kept generating and evaluating new candidates to update the pool. To create a new generation, the algorithm randomly picked two parent models from the pool, executed an unbiased parameter crossover, and applied a dynamic mutation to individual parameters. If a new candidate had a lower cost than any member of the existing pool, it replaced that member. For each generation, there were 30 new candidates being generated and evaluated. Iterative generation and evaluation of new candidate models resulted in an improved mean pool performance and convergence to an optimal mean cost (see Supplemental Materials for additional details).

The cost functions were based on the STN neuron dynamics at rest and with varying intracellular currents injected into the soma (Table 2). These features included the input resistance measured at the soma compartment, shape of the action potential, spontaneous firing frequency, F-I curve, and the hyperpolarization membrane potential with –0.1 nA current injection in soma. The experimental recordings we used to define our optimization targets were extracted from the available literature. These experiments were performed on rodent brain slices under in vitro conditions and the synaptic inputs were blocked by drugs in the ACSF (30–34). All of our model simulations were performed at body temperature (37°C). The original GW model ion channel dynamics were fitted by in vitro recordings under various temperatures and Q10 values were used to modify the rate of kinetic equations for the simulation temperature. Detailed Q10 values can be found in the code provided or in the appendix of the original GW model publication (4).

**Table 2.** Electrophysiological features of rat STN neurons

Categories	Feature	Target	Weight
Input resistance (4, 30)		50–250 MΩ	100
Spontaneous spiking properties (31–34)	Spontaneous firing	10–20 Hz	100
	Baseline potential	−65 to −55 mV	50
	Afterhyperpolarization	−75 to −60 mV	50
	Peak of action potential	10–20 mV	50
Depolarization properties (F-I curve) (32–34)	Firing frequency with a 0.04 nA current	26–36 Hz	100
	Firing frequency with a 0.1 nA current	65–75 Hz	100
	Firing frequency with a 0.16 nA current	116–126 Hz	100
Hyperpolarization properties (32)	Minimum hyperpolarizing potential with a −0.1 nA intracellular current	V_min_ ≤ −80 mV	100
	Midpoint potential of the sag	V_mid_ ≤ (V_end_ – V_min_)/2 + 1 mV	50
	Endpoint potential of the sag	V_min_ + 4 mV ≤ V_end_ ≤ V_min_ +10 mV	50

STN, subthalamic nucleus.

Typically, a rat STN neuron input resistance ranges between 100 and 200 MΩ (30), and in vitro data measured by different groups can vary substantially (4). Therefore, we extended the lower and upper limits by 50 MΩ. The cost was 0 if the model’s input resistance was within 50–250 MΩ. If the value fell outside this range, the cost was calculated using equations provided in Supplemental Materials section IV. The target value was then set to either 50 or 250 MΩ depending on whether the model’s resistance was < 50 or > 250 MΩ, respectively. Another common electrophysiological feature of rat STN neurons is that they fire spontaneously without synaptic input during in vitro slice recordings (30–37). In most cases, the observed spontaneous firing rate at body temperature (37°C) is at 10–20 Hz (31–34, 38). The cost was 0 if the model’s spontaneous firing rate was within this range.

**Figure 3.**
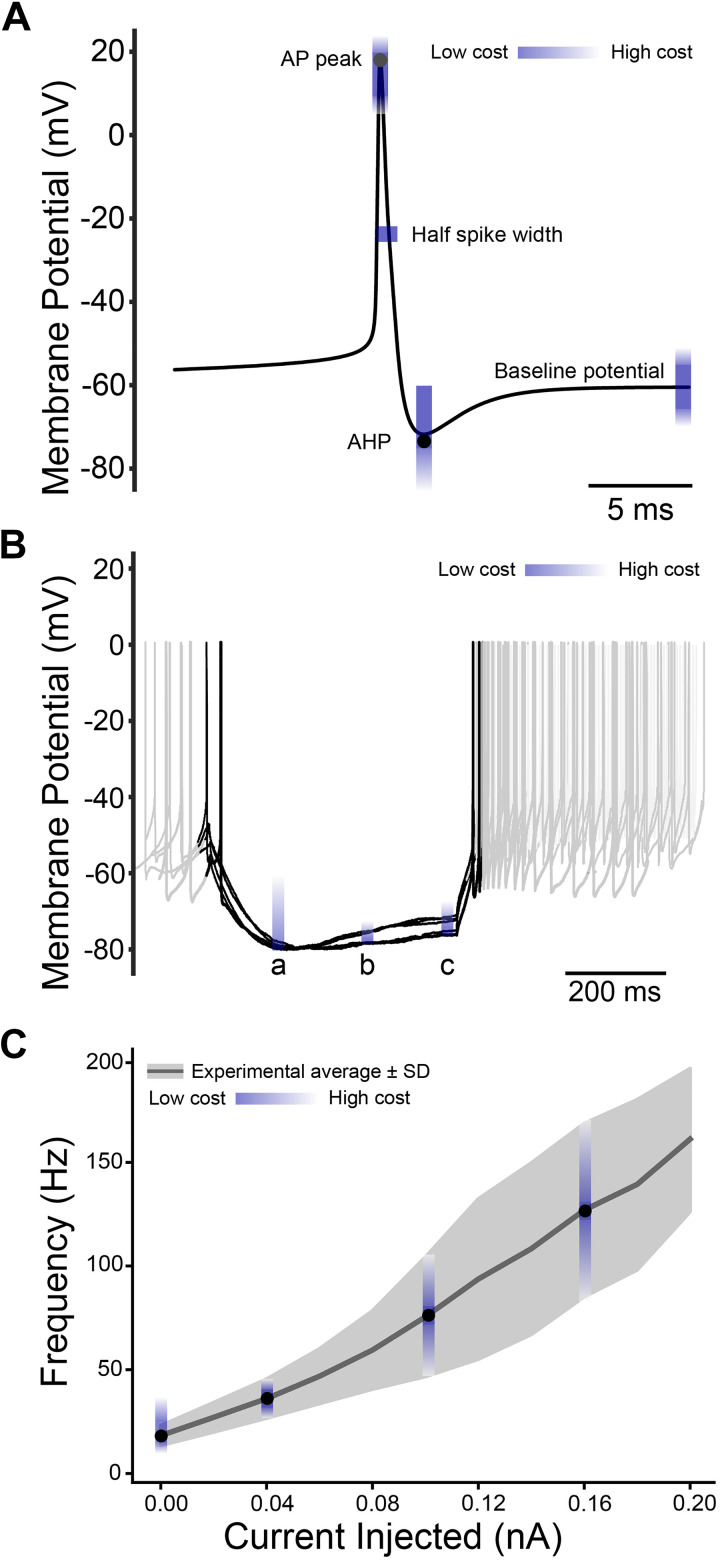
Cost function ranges. The cost range is indicated in blue color, where darker means lower cost and lighter color means higher cost. *A*: single action potential simulated by the model. Features in the cost function included AP peak amplitude, half spike duration, AHP, and baseline potential. *B*: several in vitro rat STN slice recordings when hyperpolarization currents were injected (34). Point (a) checks the minimum potential, point (b) checks the midway potential, and point (c) checks the ending potential of the sag. *C*: the experimental average of F-I curve derived from several in vitro rat STN slices recording studies (30, 34, 35). Four checkpoints at 0, 0.04, 0.1, and 0.16 nA current injections were used in the cost function. AHP, afterhyperpolarization potential; STN, subthalamic nucleus.

To evaluate the shape of the action potential, the baseline and the afterhyperpolarization potential (AHP) were scored by the cost functions. As shown in Fig. 3*A*, the baseline potential target ranged from –65 to –55 mV; the AHP target was set between –75 and –60 mV; and the peak of the action potential was set to the range from 10 to 20 mV (Table 2). In addition, the half spike width of the action potential was controlled to be less than 1 ms. The peak of an action potential was targeted in a range between 10 and 20 mV. The target values and ranges mentioned above were derived from published in vitro recordings performed on rat STN slices at body temperature (5).

Another electrophysiological feature we used was the minimum membrane potential when a negative current of 0.1 nA was injected into the soma (Fig. 3*B*). Most in vitro recordings reach −80 mV with a smaller than −0.1 nA current injected in the slice. Therefore, we set the target minimum membrane potential [point (a) in Fig. 3*B*] to be below –80 mV with −0.1 nA current injected in the soma. To control the shape of the hyperpolarization sag, the midpoint potential [point (b)] was set to be no more than 1 mV above the half amplitude of the sag and the endpoint potential [point (c)] was set to accept a range of 4–10 mV higher than the minimum membrane potential (Table 2).

The action potential firing frequency as a function of the intracellularly injected current (F-I curve) was used to assess the performance of each candidate model. The target F-I curve was derived from a mean F-I curve for rodent STN neurons obtained from three different studies (32–34). We captured the dynamics of the F-I curve in our cost function by evaluating the model’s firing frequency at 0.04, 0.1, and 0.16 nA (Table 2). The target frequency ranges were set to 26–36 Hz at 0.04 nA, 65–75 Hz at 0.1 nA, and 116–126 Hz at 0.16 nA (Fig. 3*C*).

The various features examined by the cost function (Table 2) can be divided into three major categories (spiking properties, depolarization properties, and hyperpolarization properties). The overall weight of each category was kept similar for more efficient optimization and better results. Finally, the weighted sum of these individual costs gave the total cost, which was compared with the existing pool members’ costs.

### Compatibility Check and Detailed Morphologies

Following optimization of the STN neuron model membrane properties, a final pool of 120 members was generated. Given the satisfactory performance of these 120 members under our cost function, we proceeded to randomly sample an additional 120 parameter sets within the range of the 95% confidence interval of the optimized pool. We then compared the model performance of the original optimized pool with that of the randomly sampled pool to gain a better understanding of the model's adaptability within the range provided by the optimization algorithm.

To assess how the model performed under morphological variability, we applied the updated biophysics to a collection of detailed three-dimensional (3-D) STN neuron reconstructions. The STN neuron reconstructions were created by Chu et al. (39), and we used their STN neuron control group morphologies available at NeuroMorpho.org. The dendritic lengths of these mouse STN neurons were scaled by a factor of two to adapt them into rat-sized STN neurons. This scaling resulted in average and maximum dendritic path lengths that were consistent with the original GW morphology and in line with established ranges for rat STN neurons (40). When applying the optimized biophysics to these detailed morphologies, we defined the proximal dendrites as those located ≤ 180 µm from the soma (i.e., same definition we used when updating the GW model).

## RESULTS

The goal of this study was to create an anatomically and biophysically realistic rodent STN neuron model. We started with the original GW model, added an axon, updated the ion channel distributions, and optimized their biophysics using several common electrophysiological features as our targets for replication. After three independent optimization runs with the genetic algorithm, we arrived at three final pools of model parameters. The different runs gave us very similar result ranges for each open parameter. Sensitivity analyses were then performed for *pool 1* and those details are presented in the Supplemental Material. The sensitivity analyses highlight that the sodium, fast potassium, and t-type calcium channels had the largest effects on our cost function. Those channels also have a major influence on the spontaneous firing frequency of the model. The updated model generally used lower ion channel densities [e.g., Kv3.1, KDR, sKCa, Na, and Ih (Supplemental Fig. S2)] than the original GW model. Finally, we tested the robustness of the optimized biophysics by applying them to 26 different STN neuron morphologies and examined their electrophysiological behavior. Those analyses demonstrated that the optimized biophysics were able to accommodate the anatomical variability of STN neurons.

### Spontaneous Firing Characteristics

**Figure 4.**
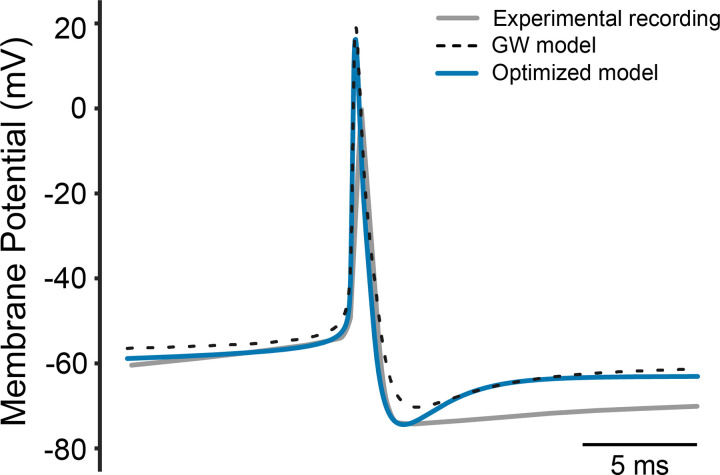
Single action potential generated spontaneously by the STN neuron model and in vitro slice recording from rat STN (34). STN, subthalamic nucleus.

The updated model was able to fire spontaneously at a rate that was within the experimental range for rodent STN neurons. Figure 4 shows a single action potential generated by the optimized model relative to the original GW model. The AHP was around –74 mV and the half spike width was around 0.66 ms. The optimization algorithm successfully mimicked the action potential peak, half spike width, and AHP amplitude targets. However, the shape of the model AHP does not perfectly align with this specific experimental recording (Fig. 4). It should be noted that there is substantial variability in the shape of the AHP reported in rat STN slice recordings (30–38). Nonetheless, both the GW model and the updated STN neuron model fail to capture the slower membrane dynamics responsible for shaping the AHP.

**Figure 5.**
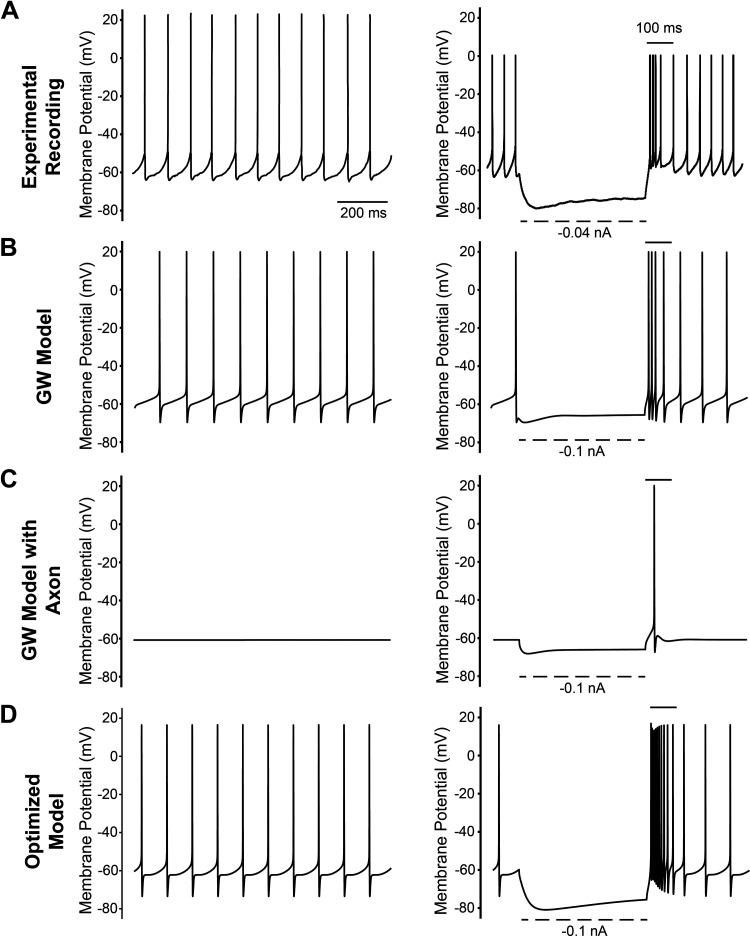
Spontaneous firing and hyperpolarization. Spontaneous firing (left column) and response to hyperpolarization (right column) from in vitro recordings (*A*) (34, 39), GW model (*B*), GW model with axon (*C*), and optimized model (*D*). GW, Gillies and Willshaw.

Figure 5 provides side-by-side comparison of the spontaneous firing characteristics of a rat STN neuron from in vitro recordings (38), the GW model, the GW model with axon, and the optimized model. Simply adding an axon to the original GW model substantially altered its electrophysiological behavior and eliminated its spontaneous firing. After optimization, the updated STN neuron model fired spontaneously at 10 Hz, which is a bit slow, but still within the experimental range. Spiking rates for the example in vitro recording and original GW model were 11 and 10 Hz, respectively (Fig. 5).

### Hyperpolarization Characteristics

STN neurons exhibit a high sensitivity to hyperpolarizing currents (Fig. 5, right column). The example in vitro recording demonstrates a membrane potential drop below –80 mV upon the injection of a –0.04 nA hyperpolarizing current. In contrast, the GW model only reached –70 mV at –0.1 nA and required twice that amount of current to reach –80 mV (4). Through model optimization, this electrophysiological characteristic was improved, enabling the updated model to reach –80 mV at –0.1 nA. The shape of the hyperpolarization sag in the optimized model also better aligned with the experimental recordings under –0.1 nA current injection.

The optimized model exhibited a notable rebound burst following cessation of a hyperpolarization current (Fig. 5). The rebound burst was generally consistent with experimental STN recordings. However, the duration of the rebound burst from the example experimental recording was ∼100 ms (32), while the optimized model exhibited a rebound burst duration of ∼150 ms.

### Depolarization Characteristics

**Figure 6.**
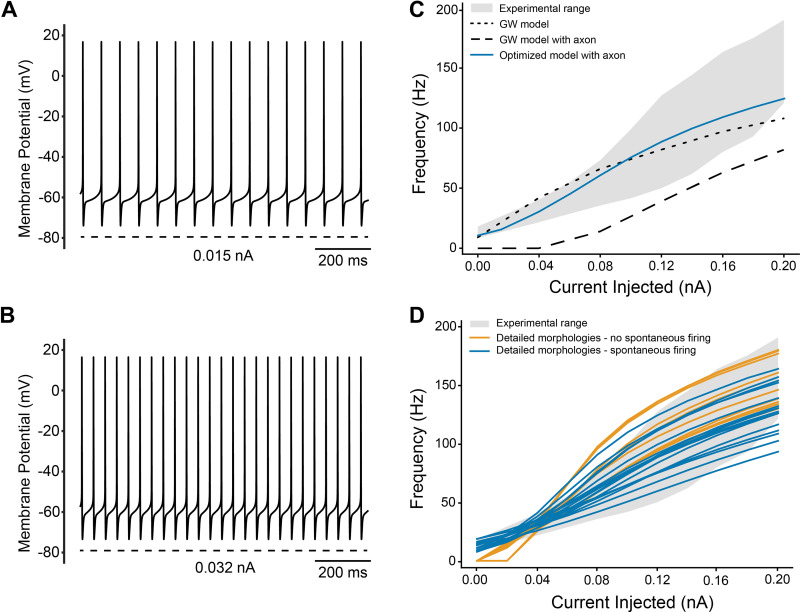
STN model response under depolarization. *A*: the model spiking at 15 Hz under 0.015 nA current injection. *B*: the model spiking at 25 Hz under 0.032 nA current injection. *C*: the frequency and current relationship of different models when depolarizing current is injected in the soma compartment. *D*: the frequency and current relationship of the detailed morphologies. GW, Gillies and Willshaw; STN, subthalamic nucleus.

When depolarizing current is injected into the soma compartment of a model it exhibits an elevated firing frequency. For our updated STN neuron model, the spiking frequency was raised to 15 Hz with 0.015 nA current injection and 25 Hz with 0.032 nA current injection (Fig. 6, *A* and *B*). Experimental recordings of STN neurons typically display a slightly sigmoidal-shaped F-I curve (32–34). Figure 6*C* presents the F-I curves of the original GW model, the GW model with an axon, and the optimized model, along with the mean F-I curve ± SD (represented by the gray-shaded area) obtained from experimental recordings. The original GW model with an axon lacked spontaneous firing and required a minimum depolarizing current of 0.04 nA to fire. However, after parameter optimization, the firing frequency of the updated STN neuron model aligned more closely with the experimental F-I range.

### Model Compatibility with Different Parameter Sets

The STN model results presented above were from 1 example “parameter set” from the optimization pool. However, the genetic algorithm approach actually generates many different example parameter sets with similar levels of fitness. To test the robustness of those different parameter set ranges, we evaluated the model’s compatibility by randomly selecting parameter sets from the 95% confidence interval of the optimized pool (Supplemental Fig. S5). For example, a random collection of ion channel conductance values (albeit derived from their channel-specific confidence intervals) was able to perform at a high level. This is somewhat remarkable and highlights the biophysical robustness of the updated STN neuron model even beyond the 120 parameter sets explicitly defined in the pool.

### Model Compatibility with Different Morphologies

**Figure 7.**
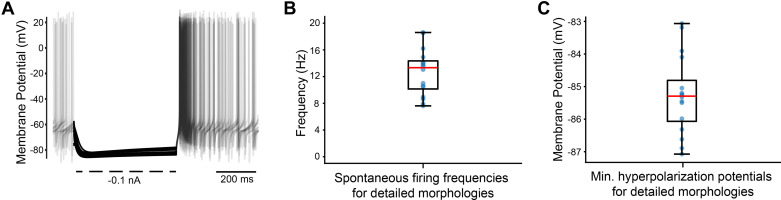
Impact of using detailed morphologies. *A*: summary plot of the hyperpolarization response (–0.1 nA) of the 16 morphologies that exhibited spontaneous firing to the optimized parameters with no modification to the sodium conductance. *B*: the average spontaneous firing rate of the 16 morphologies was 12.6 Hz (±3.4 Hz). *C*: the average minimum potential of the hyperpolarization of the 16 morphologies was –85.3 mV (±1.2 mV).

STN neurons exhibit a variety of morphologies. However, few, if any, previous modeling studies have addressed the impact of morphology on the electrophysiological behavior of STN neurons. Therefore, we examined how different morphologies influenced the performance of the optimized biophysics in reproducing our targeted electrophysiological features. Figure 6*D* provides the F-I curves obtained with each of the detailed morphologies, all using identical biophysics. The diversity in the F-I curves highlights the important role of neuron morphology in shaping the firing response to various stimulation conditions. Among the 26 detailed morphologies we tested, 16 exhibited results that fell within the acceptable range used for our optimization algorithm without any adjustments or modifications (Fig. 6*D*). Among those 16 morphologies, the model generated an average spontaneous firing frequency of 12.6 Hz (±3.4 Hz), which also fell within our target (Fig. 7*B*). In addition, the detailed morphologies successfully maintained a proper hyperpolarization potential when a current of –0.1 nA was injected (Fig. 7*A*). The average minimum hyperpolarization potential was –85.3 mV (±1.2 mV), which was below our –80 mV threshold (Fig. 7*C*). We found that a simple fix to the compatibility issue with the other morphologies was a 50% increase in the soma-dendritic sodium channel conductance. This generic adjustment allowed another 9 morphologies (i.e., 25 out of 26) to also fulfill all of our basic requirements.

**Figure 8.**
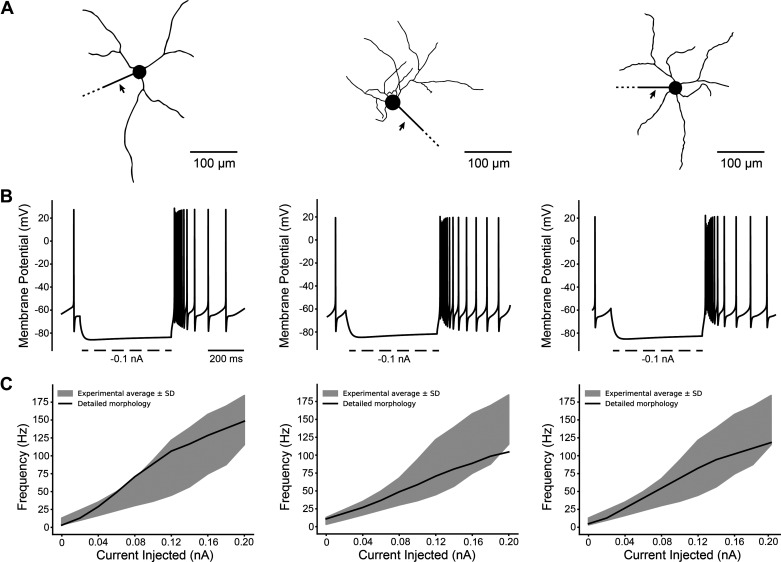
Three examples of detailed morphologies and their firing behavior. *A*: 3-D reconstructions of the neurons. Axons (indicated by arrows) added to the reconstructions were the same as used in the GW morphology. Only partial lengths of the axon are displayed for space. All three models used identical biophysical parameters. *B*: hyperpolarization characteristics for each morphology. *C*: F-I curves for each morphology. GW, Gillies and Willshaw.

Figure 8 provides three examples of detailed morphologies along with their simulated electrophysiology. Notably, all of these examples exhibited spontaneous firing. The AHP observed in these examples was consistently larger than those observed in the optimized model with the original GW soma-dendritic morphology. The Supplemental Materials provide additional details on the model performance when using different morphologies.

## DISCUSSION

The goal of this study was to update a detailed multicompartment rat STN neuron model to improve its anatomical and biophysical realism. The conductance parameters of the model were optimized using a genetic algorithm, resulting in the ability to replicate several common electrophysiological features of STN neurons (Figs. 5 and 6). We then applied the optimized biophysics to 26 different detailed STN neuron morphologies. Without requiring substantial biophysical modifications, 25 out of the 26 different STN neuron models exhibited electrophysiological characteristics that were in line with the experimental data ranges (Figs. 6, 7, and 8).

Evolutionary algorithm approaches, a superset that includes genetic algorithms, have been commonly used for tuning all kinds of neuron model parameter sets (15–17). In this study, we used a genetic algorithm and tailored the cost functions specifically to the goals of optimizing an STN neuron model. However, the algorithm used in this study can theoretically be applied to other neuron types as well. We see the genetic algorithm strategy as generally valuable in scenarios where the target neural features for replication are derived from a diverse pool of experimental datasets. This is because the algorithm works to enable adherence to the experimental range for a large population of neuron model variants. In turn, the methods are capable of defining a diverse pool of model neurons, which may be more representative of the natural biological variability in the neural population.

Computational neuroscience modeling efforts have traditionally tended to focus on using a “canonical” model to represent a given neuron type. Obviously, this kind of assumption helps simplify the modeling implementation and analysis. However, it can also result in “brittle” representations of electrophysiological features in the neural population (i.e., the model fails when biophysical and/or morphological parameters are adjusted to mimic biological variability). Thankfully, our updated “canonical” STN neuron model biophysics, derived from the genetic algorithm, performed surprisingly well in response to changes in either the neuron biophysics (Supplemental Figs. S3 and S5) or morphology (Figs. 6, 7, and 8).

Our updated rodent STN neuron model offers an improved representation of STN neuron characteristics (Fig. 5). This was achieved by explicitly incorporating an axon, refining the ion channel distributions, and optimizing ion channel conductances based on a comprehensive set of electrophysiological characteristics of STN neurons. We propose that this tool will be relevant for use in the analysis of STN firing behavior during intrinsic synaptic influences, or extrinsic electrical stimulation from intracellular or extracellular sources.

Possibly one of the most important updates in the optimized model is the inclusion of an axon. The axon plays a crucial role in transmitting information to target neurons and serves as an essential building block in a neural circuit. Moreover, extracellular electrical stimulation initiates action potentials in the axon, which then propagate both orthodromically and antidromically along the axon (3). As a result, the updated model empowers investigations into network-level questions, including the interactions between STN neurons and other basal ganglia nuclei (41).

STN neurons generally receive synaptic inputs from two primary sources, glutamatergic input from the cortex and GABAergic input from the pallidum (34). The excitatory inputs primarily target the distal dendrites (42), whereas the inhibitory inputs are distributed throughout the cell, with a higher concentration on proximal compartments (43). In addition, active and passive electrical properties, dendritic morphology, and the timing of afferent input all influence the integration of synaptic inputs. Therefore, detailed multicompartment cable models represent useful tools for studying the impact of synaptic input patterns, strengths, and locations on STN neuron firing dynamics.

The updated model will also find utility in the study of how extracellular electric fields influence STN neurons, which is particularly relevant to studying the effects of STN DBS (3). In addition, these kinds of field-cable models enable analysis of how various stimulation parameters (such as pulse duration, amplitude, and frequency) (44, 45) impact the neural response to DBS. Recent studies in patients with PD have also demonstrated that STN DBS evokes a unique oscillatory evoked potential (46–49). However, the biophysical origin of this signal remains unknown. We propose that detailed computational models will prove useful in dissecting how different neural elements, and their synaptic connections, work together to create these clinically useful evoked potential signals (50).

As shown in the sensitivity analyses (Supplemental Fig. S3), most of the ion channels were constrained by the optimization process. However, channels such as CaN, CaL, and sKCa did not have a significant effect on our cost function performance. Among these three ion channels, CaL and CaN had the least effect on our cost functions. The sKCa channel did have an effect on the neuron’s spontaneous firing rate and excitability, which is consistent with experimental analyses (32, 34). Despite the limited influence on our cost function in this study, we do not recommend removing the CaN, CaL, and sKCa channels from the model, as they might be important for other aspects of model performance that we did not examine, such as synaptic integration.

Although our optimized model improved the anatomical and biophysical realism, certain aspects of the model did not fully meet our expectations. For example, the firing frequency of the optimized model at 0.16 nA was 108 Hz, which is at the lower end of experimental range for this injected current (32–34). This discrepancy resulted from a reduced slope of the F-I curve at higher currents. One key limitation in our optimization process was that we elected to maintain all channel dynamics as per the original GW model, and our optimization only updated the channel densities. However, channel dynamics can play a pivotal role in regulating neuronal firing characteristics. For example, sodium channels strongly influence the F-I curve, and it is possible that we could have improved our match with the F-I curve with updates to the sodium channel dynamics. In addition, the current STN model consists of only one active sodium channel, while there is evidence that multiple types of sodium channels exist in STN neurons (38). It would also likely be beneficial to expand the optimization process to include the dynamics of the axonal currents, rather than focusing exclusively on the biophysics of the soma and dendrites, as the axon likely plays an important role at the higher end of the F-I curve.

Another issue with our updated STN model is that the shape of the AHP does not match the experimental data well. One possible reason could be the channel dynamics of the sKCa channel, which are known to significantly influence AHP shape (32). We attempted to explore this issue by running additional optimizations that opened up new parameters including calcium accumulation and sKCa ion channel time constants. Despite these efforts, the AHP shape remained unchanged, and the reduced slope of the F-I curve at higher currents did not improve, indicating that further modifications to the channel dynamics may be necessary to fix the problem. In addition, the precise distribution of all the different ion channels in STN neurons remains unknown. This prompted us to rely on generalized ion channel distribution characteristics. Therefore, optimizing the biophysics of the axon and time constants of all ion channels, as well as incorporating a more accurate distribution of the ion channels, could potentially translate into a more comprehensive representation of STN neuron behavior. Unfortunately, this would come at an exceptionally higher computational cost (i.e., many orders of magnitude) and likely just be an exercise in model overfitting unless a far greater level of experimental data controls on the specific ion channel biophysics were available to constrain the search.

## DATA AVAILABILITY

The updated STN neuron model and code are available for download at https://github.com/jimmychj/STN-Neuron.git. All other data and files are available on request from the authors.

## SUPPLEMENTAL DATA

10.6084/m9.figshare.24790776.v3 Supplemental Figs. S1–S6: https://doi.org/10.6084/m9.figshare.24790776.v3.

## GRANTS

This work was supported by a grant from the National Institutes of Health (R01 NS119520).

## DISCLOSURES

C.C.M. is a paid consultant for Boston Scientific Neuromodulation, receives royalties from Hologram Consultants, Neuros Medical, Qr8 Health, Ceraxis Health, and is a shareholder in the following companies: Hologram Consultants, BrainDynamics Surgical Information Sciences, CereGate, Cardionomic, Enspire DBS. None of the other authors has any conflicts of interest, financial or otherwise, to disclose.

## AUTHOR CONTRIBUTIONS

H.C., M.S.N., C.S.B., and C.C.M. conceived and designed research; H.C. and C.S.B. performed experiments; H.C. and M.S.N. analyzed data; H.C., M.S.N., and C.C.M. interpreted results of experiments; H.C. and M.S.N. prepared figures; H.C., M.S.N., C.S.B., and C.C.M. drafted manuscript; H.C., M.S.N., C.S.B., and C.C.M. edited and revised manuscript; H.C., M.S.N., C.S.B., and C.C.M. approved final version of manuscript.
